# The influence of dexmedetomidine added to ropivacaine for transversus abdominis plane block on perioperative neurocognitive disorders after radical colorectal cancer surgery: randomized, double-blind, controlled trial

**DOI:** 10.1186/s12871-024-02569-8

**Published:** 2024-05-25

**Authors:** Li Yang, RongFei Xiong, XingQu Chen, Shu Wang, DeShui Yu

**Affiliations:** 1 Department of Anesthesiology, The Second People’s Hospital of Yibin, Yibin, China; 2 Department of Rehabilitation Medicine, The Second People’s Hospital of Yibin, Yibin, China; 3 Clinical Research and Translational Center, Second People’s Hospital of Yibin City-West China Yibin Hospital, Sichuan University, Yibin, China

**Keywords:** Dexmedetomidine, Nerve block, Abdominal muscles, Perioperative neurocognitive disorders

## Abstract

**Objective:**

Perioperative Neurocognitive Disorders (PND) is a common neurological complication after radical colorectal cancer surgery, which increases adverse outcomes. So, our objective is to explore the influence of dexmedetomidine added to ropivacaine for transversus abdominis plane block (TAPB) on perioperative neurocognitive disorders, and to provide a new way to reduce the incidence of PND.

**Methods:**

One hundred and eighty patients submitted to radical laparoscopic colorectal cancer surgery were randomly divided into Control group and Dex group. Ultrasound guided TAPB was performed after anesthesia induction: 0.5% ropivacaine 20 ml was injected into each transversus abdominis plane in Control group, 0.5% ropivacaine + 1 μg/kg dexmedetomidine (amounting to 20 ml) in Dex group. We observed the incidence of PND within 30 days after surgery.

**Results:**

One hundred and sixty-nine cases were finally analyzed, including 84 cases in Control group and 85 cases in Dex group. Compared with Control group, there was no significant difference in terms of the incidence of PND on the 3rd day and the 7th day *(P* > *0.05*), but the incidence significantly decreased at the 6th hour, at the 24th hour and on the 30th day after surgery (*P* < *0.05*) in Dex group.

**Conclusion:**

Dexmedetomidine added to ropivacaine for TAPB can reduce the incidence of PND in the first 24 h after surgery and on the 30th postoperative day, which may be related to reduce the consumption of general anesthetics and provide satisfactory postoperative analgesia.

**Trial registration:**

29 /05/ 2021, ChiCTR2100046876.

## Background

Colorectal cancer is the second leading cause of cancer death in the United States, and radical resection is the most commonly used and effective procedure for it [1]. Patients often suffer from perioperative neurocognitive disorders(PND) after this procedure, which increases morbidity, mortality and the economic burden. PND was known as postoperative cognitive dysfunction(POCD) previously [2], which is related to age, general anesthetic, surgery and postoperative pain [3–6]. At present, there is no effective treatment for PND. Previous studies have shown that nerve block or dexmedetomidine can significantly reduce the incidence of PND [7, 8]. Therefore, we speculate that the combination of nerve block and dexmedetomidine may confer greater efficacy in preventing PND. Professor Brummett [9] found that dexmedetomidine added to ropivacaine for perineural nerve block can prolong the duration of analgesia and enhance the analgesic efficacy of ropivacaine. So, we wondered whether this effect is the possible mechanism that reduce the incidence of PND. Transversus Abdominis Plane Block (TAPB) is a new regional nerve block which is beneficial to the analgesia of abdominal surgery [10, 11]. Therefore, we designed this randomized, double-blinded and controlled trial to explore the influence of dexmedetomidine added to ropivacaine for transversus abdominis plane block on perioperative neurocognitive disorders after radical colorectal cancer surgery. If our hypothesis is substantiated by scientific inquiry, this approach will effectively mitigate the incidence of PND and the adverse outcomes of PND.

## Methods

### Sample size calculation

We used software PASS15.0 to calculate the sample size: 1-β = 0.9, α = 0.05. According to our preliminary experiment, we hypothesized the difference between Control group and Dex group as 17%, The incidence of PND in Dex group was 10%. A total of 172 patients were required. But some patients may not successfully complete the study. Taking this into account, the total number of patients were 180.

### Participants

This research was approved by the Ethics Committee of The Second People's Hospital of Yibin City. We used software SAS 9.4® to generate random numbers. One hundred and eighty patients submitted to laparoscopic radical resection of colorectal cancer were randomly divided into Control group (*n* = 90) and DEX group (*n* = 90) before surgery by the random numbers.

All patients signed informed consent. Briefly, we enrolled patients at the age of 60 years old or above, whose Barthel Index [12] (A tool to assess activities of daily living) scored 95 or above. We excluded patients if they were drug users, or who had regular binge drinking history (within three months), or diagnosed with cognitive dysfunction or mental disabilities. We also excluded participants if they were diagnosed with coagulation dysfunction and diabetes. All eligible patients were performed bilateral TAPB with ultrasound guided after induction by the same anesthesiologist who didn’t participate in data collection. We announced the withdrawal of participants who were operated less than 2 h, or turned to laparotomy operation during the surgery, or transferred to intensive care unit (ICU) after surgery.

### Data collection

Baselines: age, body weight, body mass index (BMI), sex, education background, and The Mini-Mental State Examination (MMSE) [13] score were obtained before surgery. Primary outcomes: the incidence of PND, which was assessed by MMSE at the 6th hour, at the 24th hour, on the 3rd day, on the 7th day and on the 30th day after surgery. Comparing with the MMSE scores before operation, if the figure decreased by more than 2 points [14], the patient shall be diagnosed with PND. Secondary outcomes: Intraoperative information (duration of operation and anesthesia, consumption of general anesthetics, fluid intake, urine and blood loss), Visual Analog pain scores (VAS) in the rest at the 6th hour, at the 24th hour, on the 3rd day, and on the 7th day after surgery. All data were collected by a blind investigator who did not know the intervention.

### Intervention

**Fig. 1:**
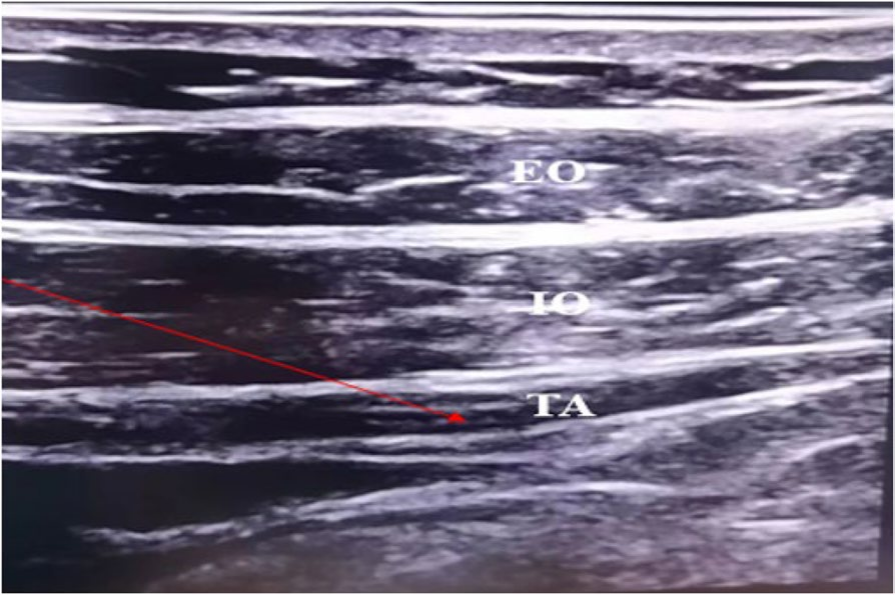
EO, externus obliquus abdominis. IO, internus obliquus abdominis. TA, Transversus abdominis

All participants, prepared preoperative fasting according to the guidelines [15], were randomly divided into Control group and Dex group by the random numbers. Both two groups of the participants were performed anaesthesia induction with the same proposal before being performed TAPB: sufentanil 0.30 μg·kg^−1^, midazolam 0.04 mg·kg^−1^, cisatracurium 0.15 mg·kg^−1^ and etomidate 0.20 mg·kg^−1^. We used lateral approach [16] with ultrasound technology to implement bilateral TAPB (Fig. 1).The only difference between the two groups was the local anesthetics of TAPB: 0.5% ropivacaine 20 ml was injected into each transversus abdominis plane in Control group, while 0.5% ropivacaine + 1 μg/kg dexmedetomidine (amounting to 20 ml) in Dex group. The remifentanil (0.05 ~ 0.3 μg·kg^−1^·min^−1^) and propofol (2 ~ 6 mg·kg^−1^·min^−1^) were continuously intravenously pumped to maintain anesthesia depth (Bispectral Index 40 ~ 60). Cisatracurium was administered (0.10 ~ 0.15 mg·kg^−1^·h^−1^) to maintain satisfactory muscle relaxation. Vasoactive drugs were used to maintain intraoperative blood pressure and heart rate fluctuations within about 20% of the participants’ baseline. The surgeon closed the incision with stitches were regarded as the operation is finished. Patients were sent back to the ward after surgery, and were given 100 mg tramadol intravenously for analgesia on time according to the degree of postoperative pain within 7 days after surgery.

### Statistical analysis

Statistical software SPSS 19.0 and Graphpad Prism7.0 were used to describe and analyze the statistical results. Data were described by mean ± standard deviation (‾*x* ± *s*), median and range interquartile(IQR). T-tests, Chi-square test and Wilcoxon rank sum test were used to analyze the differences between the two groups. A value of *P* < 0.05 was considered a significant difference.

## Results

### Baseline characteristics

**Fig. 2:**
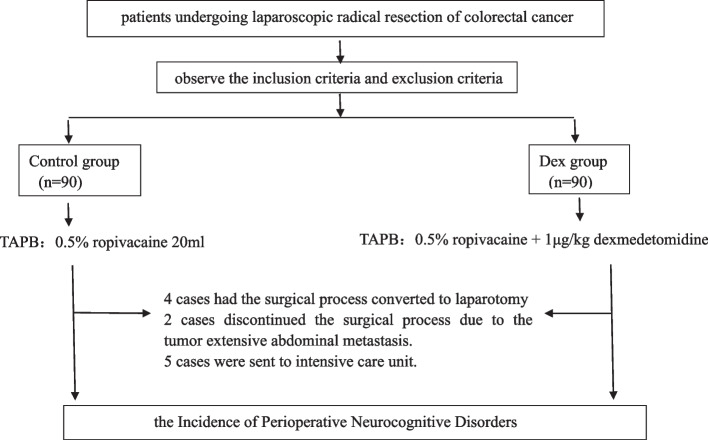
The flowchart of the trial. TAPB, Transversus Abdominis Plane Block

One hundred and eighty patients were enrolled in this trial from January 2022 to December 2022. 4 cases had the surgical process converted to laparotomy, 2 cases discontinued the surgical process due to the tumor extensive abdominal metastasis, and 5 cases were sent to intensive care unit. So, 169 cases (84 cases in Control group and 85 cases in Dex group) finally completed this study (Fig. 2).

There were no significant baseline differences between those two groups (Table 1).

**Table 1:** Comparison of basic conditions before surgery

	Control group (*n* = 84)	Dex group (*n* = 85)	t/χ2	*P*
Patient characteristics				
Age(yr)	63.61 ± 7.08	62.16 ± 6.68	1.37	0.171
Weight (kg)	59.65 ± 10.22	62.00 ± 10.29	- 1.49	0.139
BMI (kg_·_m^−2^)	23.00 ± 3.62	23.16 ± 3.33	- 0.312	0.755
MMSE	25.77 ± 1.31	25.88 ± 2.522	0.253	0.80
Sex (female/male)	34/50	27/58	1.39	0.238
Education Background			1.92	0.383
illiteracy	5(6.0)	8(9.4)		
primary	51(60.7)	56(65.9)		
≥ junior	28(33.3)	21(24.7)		

Age, weight, BMI, and MMSE are shown as mean ± standard deviation (‾*x* ± *s*), T-test. Sex, Education Background are shown as number (%), Chi-square test

### Intra-operative findings

There were no significant differences in operation types, operation and anesthesia duration, fluid intake, urine volume and blood loss between the two groups (*P* > *0.05*). However, the consumption of propofol and remifentanil in Dex group was significantly lower than that in Control group ((1.22 ± 0.42) g vs. (0.82 ± 0.33) g, (1.48 ± 0.60) mg vs. (1.17 ± 0.40) mg, *P* < *0.05*) (Table 2).

**Table 2:** Comparison of intra-operative findings

	Control group (*n* = 84)	Dex group (*n* = 85)	t/χ2	*P*
Operation type①/②	56/28	53/32	1.47	0.4793
operation(h)	4.05 ± 1.09	4.27 ± 1.16	-1.318	0.189
Anesthesia(h)	4.79 ± 1.11	4.99 ± 1.12	-1.196	0.233
Propofol (g)	1.22 ± 0.42	0.82 ± 0.33	6.77	0.000^*^
Remifentanil (mg)	1.48 ± 0.60	1.17 ± 0.40	3.87	0.001^*^
Fluid intake(ml)	2094.04 ± 533.87	2089.17 ± 545.16	0.058	0.95
Urine(ml)	429.52 ± 164.52	465.74 ± 208.48	-1.25	0.212
Blood loss(ml)	216.19 ± 138.04	244.23 ± 161.41	-1.21	0.22

①= Laparoscopic radical resection of colon cancer; ②= Laparoscopic radical resection of rectal cancer; operation (h), anesthesia (h), Propofol (g), Remifentanil (mg), Fluid intake(ml), Urine(ml) and Blood loss(ml) are shown as mean ± standard deviation (‾*x* ± *s*), T-test. Operation type ①/② are shown as number (%), Chi-square test

^*^There was a statistically significant difference between the two groups (*P* < *0.05*)

### Postoperative situation

#### Postoperative pain

**Fig. 3:**
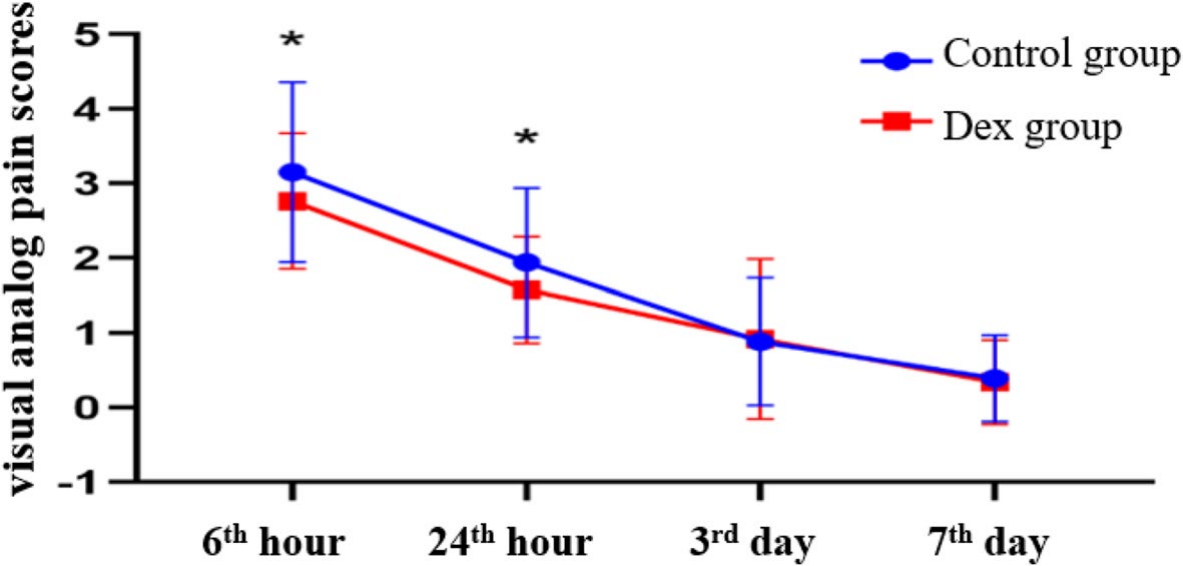
Visual analog pain scores (VAS) at rest after surgery. *There was a statistically significant difference between the two groups (*P* < *0.05*)

There was no significant difference in the VAS pain scores between the two groups on the 3^rd^ day and the 7^th^ day after surgery (*P*>*0.05*), while the VAS pain scores of Dex group at the 6^th^ hour and the 24^th^ hour after surgery were significantly lower than those of Control group (*P*<*0.05*) (Fig. 3). Analgesia frequency within 7days after surgery were significantly lower than that in Control group (*P*<*0.05*) (Table 3).

**Table 3:** Visual analog pain scores (VAS) and analgesia frequency after surgery

	Control group (*n* = 84)	Dex group(*n* = 85)	*P*	95%CI
**VAS** 6th hour	3 (2, 3.45)	3 (2, 3)	0.02*	(-0.272, 0.886)
24th hour	2 (1, 2)	2 (1, 2)	0.07*	(-0.056, 0.359)
3rd day	1 (0, 1)	1 (0, 1)	0.69	(-0.043, 0.072)
7th day	0 (0, 1)	0 (0, 1)	0.55	(-0.049, 0.040)
analgesia	1 (0, 1)	0 (0, 1)	0.04*	(-0.030, 0.032)

Visual analog pain scores (VAS) and analgesia frequency are shown as median (range interquartile, IQR), Wilcoxon rank sum test

^*^There was a statistically significant difference between the two groups (*P* < *0.05*)

#### Primary outcome: the incidence of PND

There were some patients in both groups suffering from PND within 30 days after surgery. In terms of the incidence of PND, compared with the Control group, there was no significant difference on the 3rd day and the 7th day (*P* > *0.05*), but significantly decreased at the 6th hour, on the 24th day and the 30th day after surgery in Dex group (17.9%vs.7.1%, 14.3% vs. 4.7%, 11.9% vs. 3.5%, *P* < *0.05*)(Table 4).

**Table 4:** The incidence of PND in different time after surgery

	Control group (*n* = 84)	Dex group (*n* = 85)	χ2	*P*
6th hour	15(17.9)	6(7.1)	4.527	0.033^*^
24th hour	12(14.3)	4(4.7)	4.524	0.033^*^
3rd day	7(8.3)	3(3.5)	0.995	0.319
7th day	6(7.1)	2(2.4)	1.220	0.270
30th day	10(11.9)	3(3.5)	4.170	0.041^*^

Comparing the MMSE score with that before surgery, if the figure decreases by more than 2 points, the patient shall be diagnosed with PND. Data are shown as number(%), Chi-square test

^*^ There was a statistically significant difference between the two groups (*P* < *0.05*)

## Discussion

Perioperative Neurocognitive Disorders (PND) is a common neurological complication after surgery and anesthesia, which mainly manifests as cognitive impairment. Some researchers have found that intraoperative general anesthesia may impair neurocognitive function [3, 17]. Therefore, it is our goal to reduce the consumption of general anesthetics as much as possible. In our study, there were three kinds of differences between the two groups: the consumptions of general anesthetics (propofol and remifentanil) during operation, the degree of postoperative pain and analgesia frequency, and the incidence of PND.

Firstly, the consumptions of propofol and remifentanil in the Dex group were significantly lower than that in the Control group (Table 2). We shared professor Brummett’s view [9] that dexmedetomidine added to ropivacaine for TAPB before surgery enhanced the analgesic efficacy of ropivacaine which helped to reduce the consumptions of propofol and opioid. Another reason we speculated was that the dexmedetomidine in the Dex group might be slowly absorbed into blood to produce analgesic and sedative effects. And these effects were more enduring than those caused by dexmedetomidine used continuously intravenously pumping. But it was our conjecture, we required further dynamic monitoring of the concentration of dexmedetomidine in the blood and compared the effects of dexmedetomidine used in different ways on analgesia. Secondly, VAS pain scores and analgesia frequencies of the Dex group were significantly lower than those of the Control group overall (Fig. 3, Table 3). Several studies concluded that dexmedetomidine added to ropivacaine achieved better local anaesthesia and it would not cause any major side effects in patients undergoing abdomen operation [18, 19]. And finally, the incidence of PND in the Dex group was significantly lower than that in the Control group (Table 4). We analyzed the possible reasons as follows: ①Reducing the consumptions of propofol and remifentanil. At present, some scholars believe that opiates play an important role in the occurrence of PND [20, 21]. However, the mechanism is still unclear. At the same time, the effect of propofol on postoperative cognitive function remains controversial. Laalou et al. [22] suggest that propofol can maintain natural sleep–wake cycle which may be beneficial to reduce postoperative neurocognitive damage. But another opinion [23] is that propofol has the potential to induce postoperative neurocognitive damage. So, we need further researches to confirm these findings. ②Pain. Pain and PND are closely linked through complex relationship. This relationship is relevant to the central and peripheral nervous system and cholinergic neuron. painful conditions activate the central and peripheral nervous system. And then the nervous system releases inflammatory factors, such as bradykinins, interleukins, tumor necrosis factor-α and prostaglandins, which may cause changes in cognitive function [24, 25].③ The effect of dexmedetomidine. Dexmedetomidine as a α_2_-adrenergic agonist has good analgesic and sedative effects. At present, a large number of reports have proved that dexmedetomidine, as a maintenance agent for general anesthesia, can reduce the incidence of postoperative cognitive dysfunction (POCD) in patients undergoing cardiac surgery and non-cardiac surgery [26, 27]. In the end, we found that it is worth noticing that the differences in the incidence of PND between the two groups were mainly shown at 6th hour, at 24th hour and on the 30th day after surgery. The differences observed in the early postoperative period were primarily attributed to variations in the intensity of postoperative pain and the effectiveness of TAPB analgesia. Postoperative pain in patients undergoing abdominal surgery mainly occurs at the 8th hour to the 16th hour after surgery, while dexmedetomidine added to ropivacaine has sensory block durations of 505.1 ± 113.9 min [28]. On the 30th day after surgery, the incidence of PND showed a difference again. The exercise and mood [29] of patient after surgery may make a difference in neurocognitive function. In addition, patients might review the contents of the MMSE scale, which could influence the result. therefore, we should take more factors into account in our further study.

## Limitation

First, this study was implemented in a single center. There are some shortcomings such as small sample size and narrow population coverage. One single-center experiment is prone to Berkson's bias. Besides, in terms of patients, it is thoughtless to compare the difference in type of operation (laparoscopic radical resection of colon cancer and laparoscopic radical resection). The stage of colorectal cancer should be taken into account. The stage of tumor significantly affects the postoperative complication rate, which may bring different mood. Depressed mood [29] may exert an influence on neurocognitive function. In order to resolve these problems, we need more samples, specify the stage of tumor and organize multicenter studies in our further study.

## Conclusion

Our single-center randomized controlled study shows that dexmedetomidine added to ropivacaine for TAPB can reduce the incidence of PND in the first 24 h after surgery and on the 30th postoperative day, which may be related to reduce the consumption of general anesthetics and provide satisfactory postoperative analgesia.

## Data Availability

The datasets used and analysed during the current study are available from the corresponding author on reasonable request.

## Abbreviations

BMI: Body mass index
ICU: Intensive care unit
MMSE: Mini-Mental State Examination
PND: Perioperative neurocognitive disorders
POCD: Postoperative cognitive dysfunction
TAPB: Transversus abdominis plane block
VAS: Visual analog pain scores
